# Perforation of the Pulmonary Vein During Ablation of Atrial Fibrillation: A Rare Complication of Cryoballoon Ablation

**DOI:** 10.19102/icrm.2023.14112

**Published:** 2023-11-15

**Authors:** Enes Elvin Gul, Muhammad Salman Ghazni, Hasan Sandougji

**Affiliations:** 1 Division of Cardiac Electrophysiology, Madinah Cardiac Centre, Madinah, Saudi Arabia; 2 Department of Cardiovascular Surgery, Madinah Cardiac Centre, Madinah, Saudi Arabia

**Keywords:** Atrial fibrillation, cryoablation, pulmonary vein perforation

## Abstract

A 58-year-old man admitted for a cryoballoon ablation due to a history of symptomatic paroxysmal atrial fibrillation experienced pericardial effusion and cardiac tamponade intraoperatively. A longitudinal left superior pulmonary vein perforation was confirmed by emergency thoracotomy and repaired. He developed atrial fibrillation 2 days postoperatively, which was terminated with colchicine and oral steroids the following day.

## Case presentation

A 58-year-old man was admitted for a cryoballoon (CB) ablation (CBA) due to a history of symptomatic paroxysmal atrial fibrillation (AF). Transthoracic echocardiography revealed normal left ventricular function and left atrial size (left atrial diameter, 39 mm). After discussion with the patient, a catheter ablation with CB was offered. Consent was obtained from the patient.

**Figure 1:**
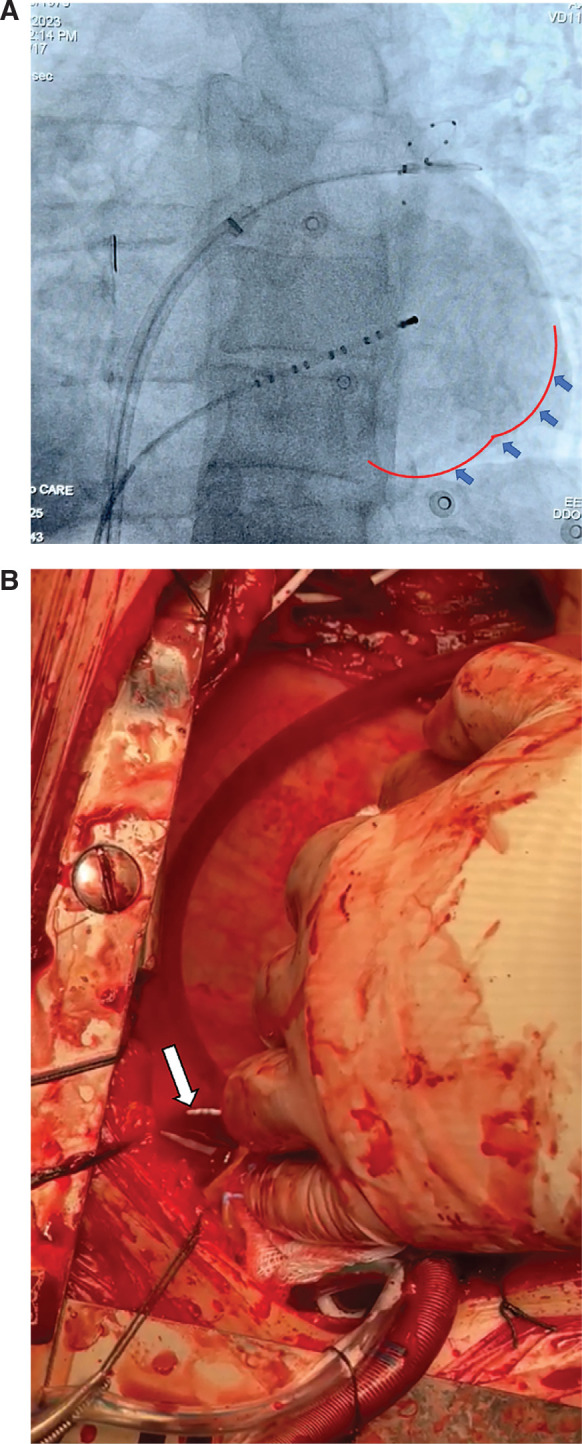
**A:** Fluoroscopic view (left anterior oblique) showing the rim of pericardial effusion (arrows). **B:** Emergency thoracotomy revealed a 1.5-2.0-cm longitudinal left superior pulmonary vein perforation (arrow).

The patient was taken to the electrophysiology laboratory. The procedure was performed under conscious sedation. Catheters were inserted and a transseptal puncture was performed with the guidance of intracardiac echocardiography (ICE). A 28-mm CBA catheter (Arctic Front Advance™; Medtronic, Minneapolis, MN, USA) was inflated at the ostium of the left superior pulmonary vein (PV) (LSPV). Venography showed complete occlusion of the vein without any residual contrast leakage. The first application for 240 s demonstrated a very good temperature drop (−58°C), and the thawing time was >15 s. In addition, clear isolation of PV signals was observed during freezing. Then, we observed residual PV signals, and the second application was completed with cannulation of the lower branch of the LSPV. This time, the temperature drop was significant, reaching −60°C within 120 s. Due to the rapid temperature drop, the application was terminated at 120 s. We subsequently noticed an immediate drop in blood pressure, and fluoroscopy in the left anterior oblique view revealed a rim of pericardial effusion **(Figure 1)**. Pericardial effusion was confirmed with ICE as well. The patient was in cardiac tamponade. Immediate percutaneous pericardial drainage was performed with uncontrollable bleeding. Therefore, the patient was immediately taken for an exploratory sternotomy. An emergency thoracotomy revealed a 2.0-cm longitudinal LSPV perforation **(Figure 1)**, which was repaired. On postoperative day 2, he developed AF with rapid ventricular response, which did not respond to medical or electrical cardioversion. He was started on colchicine and oral steroids and the AF terminated the next day. The patient was discharged home on postoperative day 4.

## Discussion

CBA has emerged as an effective treatment option to treat AF. Although CBA is a safe procedure, severe complications can occur. In addition to the traditional major complications associated with AF ablation, such as stroke, cardiac tamponade, and atrio-esophageal fistula, CBA is also associated with phrenic nerve palsy and bronchial injury.^1^ However, there are also reports revealing some rare complications of CBA, such as entrapment or perforation of the PV.^2–5^ The level of perforation may be either proximal or distal. If perforation occurs distally into bronchi, the patient might suffer from severe bleeding into the lungs and hemoptysis. There have been few cases showing PV perforation into bronchi.^6^ In our case, perforation was at the ostial level; therefore, there was no evidence of pulmonary complication.

The speculated causes of PV perforation during CBA are as follows: harsh/uncontrolled manipulation of a circular mapping catheter, intra-PV inflation and freezing of the CB, premature pulling of the CB and circular catheters before thawing has been completed, shape of the PV, and very low nadir balloon temperature.^5,7,8^

In our patient, although both inflation and freezing of CB were not performed inside the PV, we speculated that the PV perforation may have been caused by the tip of the CB catheter, which damaged the proximal part of the LSPV. Careful handling of both circular and CB catheters during CBA is very crucial.

PV perforations during CBA should only be managed surgically. Operators should be aware of this very rare complication.

## References

[1] Mugnai G, de Asmundis C, Ciconte G (2015). Incidence and characteristics of complications in the setting of second-generation cryoballoon ablation: a large single-center study of 500 consecutive patients. Heart Rhythm.

[2] Yoshizawa R, Owada S, Sawa Y, Deguchi H (2020). Successful removal of a circular mapping catheter which perforated the pulmonary vein during cryoballoon ablation by lateral thoracotomy: a case report. Eur Heart J Case Rep.

[3] Massaro RC, Burke JA, Altemose GT (2015). Fracture and retrieval of an Achieve circular mapping catheter in and from a pulmonary vein during cryoballoon ablation for atrial fibrillation. HeartRhythm Case Rep.

[4] Makimoto H, Kelm M, Shin DI, Blockhaus C (2017). Breakage of a circular catheter wedged in a right pulmonary vein during cryoballoon pulmonary vein isolation. Intern Med.

[5] Sudo K, Shigeta T, Oda A (2022). Pulmonary vein perforation and life-threatening hemoptysis during cryoballoon ablation for persistent atrial fibrillation. JACC Case Rep.

[6] Fukunaga H, Higuchi R, Tanizaki K, Isobe M (2019). Pulmonary vein perforation into bronchi: a rare but life-threatening complication of cryoballoon ablation. Eur Heart J Case Rep.

[7] Hori Y, Nakahara S, Taguchi I (2017). Roof-dependent atrial-flutter after a 28 mm second-generation cryoballoon ablation. Europace.

[8] Kulkarni N, Su W, Wu R (2018). How to prevent, detect and manage complications caused by cryoballoon ablation of atrial fibrillation. Arrhythm Electrophysiol Rev.

